# The addition of a pH-sensitive gel improves microemulsion stability for the targeted removal of colonic ammonia

**DOI:** 10.1186/1471-230X-11-50

**Published:** 2011-05-10

**Authors:** Ai-Hong Wang, Zhi-Jun Duan, Ge Tian, Dan Lu, Wen-Jun Zhang, Gao-Hong He, Gui-Hua Fang

**Affiliations:** 1 Department of Gastroenterology, First Affiliated Hospital of Dalian Medical University, Dalian 0086-116011, Liaoning Province, China; 2 College of Pharmacy, Dalian Medical University, Dalian 0086-116041, Liaoning Province, China; 3 Membrane Science and Technology Center, Dalian University of Technology, Dalian 0086-116012, Liaoning Province, China; 4 This author is currently working at Tangshan Gongren Hospital 0086-063000, Hebei Province, China

## Abstract

**Background:**

We prepared an oral W/O microemulsion for the removal of colonic ammonia (ME-RCA). The effect of this microemulsion was influenced by the digestion process in the gastrointestinal tract. In this paper, we aim to show that stability was improved by using a microemulsion-based gel for the removal of colonic ammonia (MBG-RCA).

**Methods:**

MBG-RCA was prepared by adding sodium alginate to the ME-RCA. MBG-RCA and ME-RCA were passed through a simulated gastrointestinal environment, and the amount of colonic ammonia present was then determined by titration with a standard solution of hydrochloric acid. The pH of the gastrointestinal fluid was measured using a pH test paper and the size and form of the microemulsions were examined under the microscope. 18 healthy rats were randomly divided into three groups, fasted for 24 hours and allowed to drink normally. Three-way pipes were placed at the gastroduodenal junction in Group I, and at the terminal ileum in Group II. After the intragastric administration of ME-RCA, the stomach contents in Group I, the effluent from the terminal ileum in Group II and discharge from the anus in Group III were collected. The pH values of the gastrointestinal juice were measured by the pH test paper and those of the colon were determined by a universal indicator. These animal experiments were also used to test the effect of MBG-RCA.

**Results:**

MBG-RCA showed a better removal rate of artificial colonic ammonia than ME-RCA (P < 0.05). The decrease in pH value of the artificial small intestinal fluid due to ME-RCA did not occur when MBG-RCA was used. In the simulated gastrointestinal process, MBG-RCA maintained greater stability and released the emulsion (ME-RCA) in the colonic fluid. In the gastrointestinal tract of normal SD rats, ME-RCA decreased in size and lost its stable form after entering the small intestine, while MBG-RCA remained stable and intact emulsion-drops were observed from the anus. Neither substance had any effect on the pH of the stomach or colon of normal rats (partly because normal rats were fasted for 24 hours and allowed to drink normally, which resulted in a low level of ammonia production in the colon). Unlike ME-RCA, MBG-RCA did not reduce the pH of the small intestine.

**Conclusions:**

MBG-RCA was more stable in the gastrointestinal tract and more effective at removing colonic ammonia when a higher concentration of ammonia was present. This made it possible to achieve the targeted removal of colonic ammonia and is a promising method to prevent hepatic encephalopathy (HE) in future studies.

## Background

Ammonia intoxication theory is central in the pathogenesis of hepatic encephalopathy (HE) [1]. The elimination of ammonia is the main method of preventing and treating HE [2,3]. Intestinal ammonia is mainly produced in the colon. To date, the oral methods used to eliminate intestinal ammonia have been ineffective. The main problem is that current methods have no significant way of eliminating ammonia directly and the clearance rate is low. In order to solve these problems, we prepared an oral W/O microemulsion for the removal of ammonia (ME-RCA). In accordance with the theory of acid-base balance, acetic acid was used as the water phase for ME-RCA in order to remove ammonia, an alkaline substance. In earlier experiments, it was proved that ME-RCA is effective in eliminating ammonia, however, its effect can be influenced by the digestive process in the gastrointestinal tract [4]. It has been reported by some researchers that pH-sensitive gels can swell in the colon and release drugs due to the pH gradient in gastrointestinal fluids [5,6]. Therefore, we added ME-RCA to the sodium alginate solution and prepared a microemulsion-based gel for the removal of ammonia (MBG-RCA), with the aim of enhancing its stability in the gastrointestinal tract and to achieve the colon-specific release of the microemulsion to increase ammonia elimination in the colon.

## Methods

### Animal care considerations

The experimental protocols were approved by the Animal Care and Use Committee of Dalian Medical University in accordance with guidelines established by the Canadian Council on Animal Care.

### Preparation

W/O ME-RCA and MBG-RCA were prepared by the Membrane Science and Technology Center at the Dalian University of Technology. The ME-RCA was prepared using a formulation containing Tween 80 (surfactant), ethylene glycol (cosurfactant), dimethyl silicone oil (external oil phase), and an ammonia absorbent with a solution of 30% acetic acid and 0.9% sodium chloride (internal phase). The mass ratio of surfactant and cosurfactant was 1:1, and the mass ratio of the oil and mixed surfactant was 2:1. The W/O microemulsion system contained a 20% water solution. The microemulsion was added to the sodium alginate solution at a ratio of 1:1, which was then stirred by a magnetic stirrer for 30 minutes to prepare MBG-RCA.

The artificial gastrointestinal fluid was prepared according to the Chinese Pharmacopoeia, 2005 edition [Part II List].

Pepsin (Shanghai Becky Biotechnology Co. Ltd), trypsogen (Hefei Bo Mei Biotechnology Co. Ltd), urethane (Chinese Medical Clique Chemical Reagent Company), Sudan III (Chinese Pharmaceuticals Company), and precise pH test paper (Shanghai Sanaisi Reagent Co. Ltd) were used in this study.

### The elimination of colonic ammonia and the stability of ME-RCA and MBG-RCA in a simulated gastrointestinal environment

Appropriate amounts of ME-RCA or MBG-RCA (with the same volume of ME-RCA) were added to the artificial liquid. The clearance rates of artificial colonic ammonia were measured by simulating gastrointestinal transit time and pH environment, in accordance with the method previously described [4]. Appropriate proportions (1:5) [7] of both preparations were respectively added to the artificial gastric juice and left to stand for 2 hours. These were then added to the artificial small intestinal fluid for 3 hours, and then the ammonical artificial colonic fluid (10 g/L ammonia) and left to stand for 10 hours. The ammonical lower layer was then used in the experiments. The size and form of ME-RCA and MBG-RCA were observed under the microscope and the pH values in the fluids were monitored using precise pH test paper after the fluids had been left to stand.

Ammonia in the artificial colonic fluid was measured according to international standards (ISO 6353/2-1983). The quantity of ammonia was determined by titration using a standard solution of hydrochloric acid (0.5 mol/L), and the volume of hydrochloric acid consumed was recorded. The quantity of ammonia in the colon: m = 0.5 × 0.01703 × V.

The percentage of ammonia removed:

The above experiments were performed three times.

### Stability in the gastrointestinal environment in normal SD rats

18 SD rats were randomly divided into three groups. 6 rats in each group were fasted for 24 hours and allowed to drink normally. ME-RCA (0.005 L/kg) was used for intragastric administration. Intraperitoneal injections of anesthesia (10% urethane, 1000 mg/kg) were used and surgery was carried out at 37°C in a thermostatic water bath kettle. A three-way pipe was placed at the gastroduodenal junction, and the stomach contents were collected at 10 min, 20 min, 30 min, 60 min and 120 min after emulsion administration in Group I. In Group II, a three-way pipe was placed at the terminal ileum, and the effluents from the small intestine were collected 5 hours after administration. In Group III, discharges from the anus were collected when emulsion-drops were observed. MBG-RCA (0.01 L/kg) was also used for intragastric administration in another 18 rats. Therefore, ME-RCA or MBG-RCA was investigated in different segments of the gastrointestinal tract after intragastric administration. The changes to the substances after passing through the stomach were investigated in Group I, in the small intestine in Group II, and in the whole gastrointestinal tract in Group III. After staining with Sudan III, the size and form of the collected contents in each group were observed under the microscope. The pH values of the gastric and small intestinal fluid were measured using precise pH test paper before and after intragastric administration. The pH of the colon was measured by universal indicator-cotton stick before intragastric administration and when ME-RCA or MBG-RCA was observed in discharges from the anus.

### Statistical analysis

Data analysis was performed using SPSS 13.0 software. All results are expressed as mean ± standard error of the mean. Repeated measures analysis of variance was used to test for differences in the pH of ME-RCA and MBG-RCA in the simulated gastrointestinal environment and in the stomach of rats. The two-sample t-test was used to test for differences in ME-RCA and MBG-RCA in eliminating artificial colonic ammonia. The two-sample t-test was used to test for differences in the pH of ME-RCA and MBG-RCA in the small intestine and colon of rats. P < 0.05 was considered statistically significant.

## Results

### Comparison of the effectiveness of ME-RCA and MBG-RCA in eliminating artificial colonic ammonia

After digestion of the enzyme-containing artificial gastric and small intestinal fluid, clearance rates of ME-RCA and MBG-RCA for ammonia in the artificial colonic fluid were 51.9 ± 1.85% and 55.92 ± 4.76%, respectively. The findings were statistically significant (*P *< 0.05), and the clearance rate of MBG-RCA was higher than ME-RCA.

### Comparison of the stability of ME-RCA and MBG-RCA in the simulated gastrointestinal environment

#### Size and form

**Figure 1:**
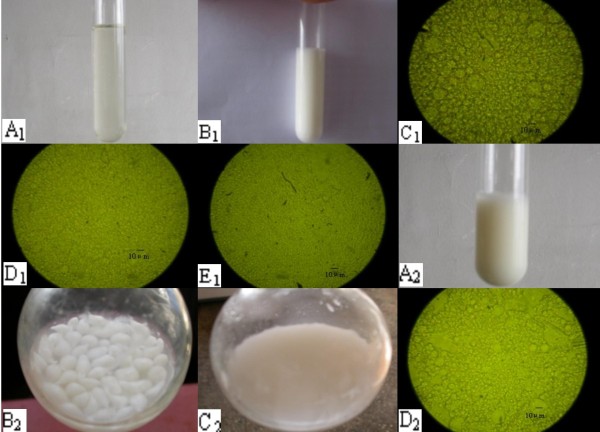
**Size and form of ME-RCA and MBG-RCA in a simulated gastrointestinal environment *in vitro***. A1 - ME-RCA translucent appearance; B1 - ME-RCA in a gastrointestinal environment; C1 - ME-RCA in artificial gastric fluid (× 100); D1 - ME-RCA in small intestinal fluid (× 100); E1 - ME-RCA in colonic fluid (× 100) A2 - appearance of MBG-RCA; B2 - MBG-RCA in artificial gastric fluid and small intestinal fluid; C2 - MBG-RCA in colonic fluid; D2 - MBG-RCA in colonic fluid under microscope (× 100) (after arriving at the stomach, the microemulsion-gel system encountered acid and became a gel)

ME-RCA was seen to swell and become an emulsion in artificial gastric fluid, and maintained a stable size in the artificial small intestinal and colonic fluid. MBG-RCA particles could be seen in the artificial gastric and small intestinal fluid, and the size and form remained stable and became emulsion-like in artificial colonic fluid. Emulsion could be seen under the microscope, which confirmed that ME-RCA was released in artificial colonic fluid (Figure 1).

#### pH value

**Figure 2:**
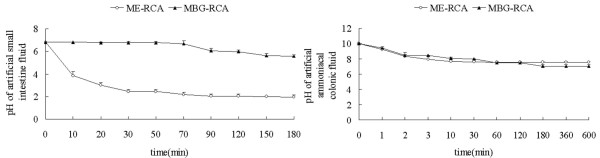
MBG-RCA significantly reduced the impact of the pH of artificial small intestinal fluid compared with ME-RCA and the influences of both on the pH of artificial ammoniacal colonic fluid

ME-RCA and MBG-RCA had no effects on the pH of artificial gastric fluid. Both decreased the pH of artificial small intestinal fluid, and MBG-RCA significantly reduced the impact of pH compared with ME-RCA (*P *< 0.05). Both decreased the pH of artificial colonic fluid (*P *< 0.05) with similar effects (Figure 2).

### Comparison of the stability of ME-RCA and MBG-RCA in the gastrointestinal tracts of normal rats

#### Morphologic changes

**Figure 3:**
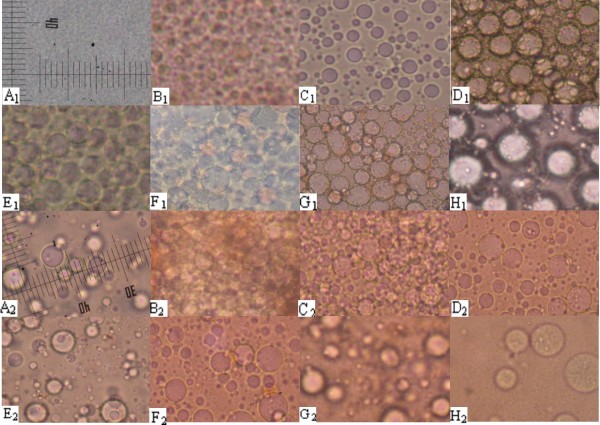
**Morphological changes of ME-RCA and MBG-RCA in the gastrointestinal tract of normal rats observed by microscopy**. A1 - Prepared ME-RCA; B1 - ME-RCA in stomach 10 min; C1 - ME-RCA in stomach 20 min; D1 - ME-RCA in stomach 30 min; E1 - ME-RCA in stomach 60 min; F1 - ME-RCA in stomach 120 min; G1 - ME-RCA in small intestine; H1 - ME-RCA in colon (× 400) A2 - Prepared MBG-RCA; B2 - MBG-RCA in stomach 10 min; C2 - MBG-RCA in stomach 20 min; D2 - MBG-RCA in stomach 30 min; E2 - MBG-RCA in stomach 60 min; F2 - MBG-RCA in stomach 120 min; G2 - MBG-RCA in small intestine; H2 - MBG-RCA in colon (× 400)

The mean diameter of ME-RCA was less than 0.1 μm. ME-RCA swelled and became an emulsion after entering the rat's stomach. The emulsion droplets gradually became larger within the first 30 min, and then remained stable. However, they were deformed after reaching the small intestine. Although the MBG-RCA preparation was not optimal, we observed that the particles maintained their integrity and had no significant changes 20 min after entering the stomach until they reached the small intestine. Integrated W/O emulsion-drop discharges were detected after passing through the colon (Figure 3).

#### Influences on gastrointestinal Ph

**Figure 4:**
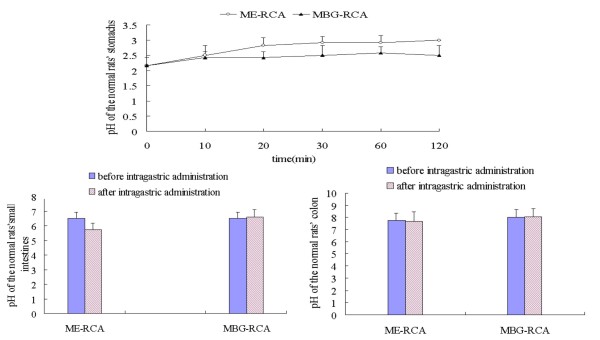
**Influence of ME-RCA and MBG-RCA on pH of the stomach, small intestine and colon of normal rats**.

The stomach pH remained unchanged when either ME-RCA or MBG-RCA entered the stomach. The pH value of the small intestine decreased in rats administered ME-RCA (*P *< 0.05) and remained stable in those administered MBG-RCA (*P *> 0.05). Neither substance had any obvious influence on rat colon pH (*P *> 0.05) (Figure 4).

## Discussion

We used a W/O ME-RCA prepared using acetic acid as a trapping agent to remove ammonia in artificial colonic fluid. The internal water phase of the emulsion was an acetic acid solution and ammonia was absorbed into the emulsion (the microemulsion swelled to the size and form of the emulsion in the colon) and neutralized by acetic acid. However, its instability in gastrointestinal juice limits its further use in research and other applications. In this study, MBG-RCA was prepared by embedding the ME-RCA in pH-sensitive gel to improve its stability.

The liquid membrane used to absorb ammonia should be stable enough and maintain its integrity while passing through the gastrointestinal tract until it is expelled out of the body [8]. The ideal preparation for the targeted elimination of ammonia in the colon should be unaffected by gastric and small intestinal juice, and maintain its integrity until it reaches the colon. Transport time and pH of the gastrointestinal tract are the main factors that affect the release speed and position of oral drugs, and the stability of liquid membrane drugs. Changes in the size and form could reflect the stability of the liquid membrane. Because the internal water phase was acetic acid, once it dissolves, the pH of the gastrointestinal juice would decrease and mucosa could be damaged. Microemulsions are more stable and have a much longer shelf life than emulsions and are often used as drug carriers *in vitro*. When taken orally, a microemulsion is diluted by the gastric juice and becomes an emulsion. This is a key problem in maintaining the stability and character of the microemulsion when it is used as a drug carrier[9].

In the simulated gastrointestinal environment, ME-RCA swelled in the artificial gastric fluid and its size and form then remained stable in the artificial small intestinal and colonic fluid. ME-RCA had no effect on the pH of the artificial gastric fluid, while it significantly reduced the pH of the artificial small intestinal fluid. We established models for the stepwise gastrointestinal investigation by placing three-way pipes at the gastroduodenal junction and at the terminal ileum and collecting discharges from the anus. Water and food could be taken normally when the rats awoke from surgery. Normal gastrointestinal transit can be maintained at a maximum and observed using this method. It was observed that ME-RCA became deformed and reduced in size, and lowered the pH of the small intestine in normal rats, while MBG-RCA obviously reduced these changes compared with ME-RCA.

The reason why ME-RCA swelled in the artificial gastric juice may be related to gastric fluid dilution, however, it did not result in a change in gastric juice pH. The reason why ME-RCA led to a decrease in pH in the small intestine may indicate a leakage of acidic substances. In order to overcome the shortcomings of ME-RCA and allow it to pass through the stomach and small intestine to successfully reach its objective in the colon, we added a natural polymer, sodium alginate, to optimize the preparation of MBG-RCA on the basis of ME-RCA. MBG-RCA has both the advantage of a microemulsion and a gel. This could enhance the stability of drugs and achieve slow-release and controlled-release of certain drugs due to its strong viscosity. Moreover, it possesses the characteristics of an intelligent gel controlled-release drug system and utilizes the response to external pH to achieve targeted colonic release of ME-RCA.

Gels may respond to temperature, pH, and ionic strength, and can achieve drug release with respect to time, quantity, or location to enhance the effects of pharmacodynamics when intelligent polymer materials are added to form intelligent gels. Drug release can be controlled by swelling and shrinkage. Some researchers have designed and synthesized a copolymerization gel system which contains not only a pH-sensitive hydrophilic compound but also a hydrophobic long-chain latent compound and an azo compound that can be degraded by bacteria. This formulation can protect the drug from damage due to gastric enzymes and control swelling of the gel in the small intestine by adjusting its composition or changing the length of the hydrophobic chain. Early release of the drug within the gel in the small intestine can then be avoided, and the drug can be protected until it is released in the colon when the gel has been degraded[5,6]. A copolymer hydrogel formed by polyvinyl alcohol and acrylic acid or methacrylic, has a protective effect on insulin in a gastric acid environment, however, it starts to release or release a larger quantity in the alkaline conditions of the intestine [10,11].

Alginic acid insolubilizes in the low pH environment of the stomach, while it dissolves in the alkaline environment of the intestine and can also be degraded by colonic enzymes. It has been widely used for the slow-release and controlled-release of drugs[12]. Some studies have shown that calcium alginate gel can remain stable in gastric juice and swell in the intestinal fluid. It is a good carrier which controls the release of materials such as acidic drugs[13]. Chen et al[14] prepared a N, O-carboxymethyl chitosan and alginic acid hydrogel with a swelling ratio of 2.5 at pH 1.2 and 6.5 at pH 7.4. Correspondingly, the quantity of encapsulated bovine serum albumin released in acidic conditions was 20%, while as high as 80% was released in alkaline conditions.

As a brand-new drug carrier, there is no available research on oral microemulsion-gels for the elimination of gut ammonia. On the basis of the studies mentioned above, MBG-RCA was prepared according to changes in the gastrointestinal tract pH, and thus had the advantage of an intelligent gel with respect to pH. We found that MBG-RCA prepared on the basis of ME-RCA was more effective at eliminating ammonia and had greater stability in the gastrointestinal tract. It successfully protected ME-RCA and could pass through the stomach and small intestine. This indicated that it would be possible to realize the objective of targeted colonic release of ME-RCA. In this study, we did not find a change in colonic pH when MBG-RCA was administered to normal rats. The former experiment showed that a higher concentration of ammonia was necessary for MBG-RCA to work well. A significant difference in the effectiveness of oral MBG-RCA and ME-RCA for eliminating colonic ammonia has not been shown in animal studies because the rat model did not include higher levels of ammonia production in the colon. In this experiment, normal rats were fasted for 24 hours and allowed to drink normally, which led to a low level of ammonia production in the colon. Despite this, the desired results were achieved. The data were sufficient to demonstrate that MBG-RCA was more stable in the gastrointestinal tract than ME-RCA.

## Conclusions

MBG-RCA maintained greater stability in the gastrointestinal tract and was more effective in the removal of colonic ammonia when a higher concentration of colonic ammonia was present. This made it possible to achieve the targeted removal of colonic ammonia and is a promising method for preventing HE in future studies. The next step in our research is to continue to optimize the composition and preparation of ME-RCA and MBG-RCA in order to improve the removal rate of colonic ammonia, as well as its stability in the stomach and small intestine and pH sensitivity in the colon. An HE animal model should be developed to investigate the targeted removal of colonic ammonia and to determine its effectiveness at preventing and treating HE.

## Competing interests

The authors declare that they have no competing interests.

## Authors' contributions

ZJD designed and modified this issue, GT, AHW, WJZ, DL and GHF designed and performed the experiments, AHW, DL and GHF completed the paper, and GHH participated in designing the issue. All authors read and approved the final manuscript.

## Pre-publication history

The pre-publication history for this paper can be accessed here:

http://www.biomedcentral.com/1471-230X/11/50/prepub
